# Definitions and elements of endpoints in phase III randomized trials for the treatment of COVID-19: a cross-sectional analysis of trials registered in ClinicalTrials.gov

**DOI:** 10.1186/s13063-021-05763-y

**Published:** 2021-11-08

**Authors:** Kentaro Sakamaki, Yukari Uemura, Yosuke Shimizu

**Affiliations:** 1 Center for Data Science, Yokohama City University, 22-2 Seto, Kanazawa-ku, Yokohama, 236-0027 Japan; 2 Department of Clinical Research, National Center for Global Health and Medicine, Tokyo, Japan

**Keywords:** COVID-19, Characteristics of endpoints, Clinical course, Time frame, Phase III trials

## Abstract

**Background:**

There are several challenges in designing clinical trials for the treatment of novel infectious diseases, such as COVID-19. In particular, the definition of endpoints related to the severity, time frame, and clinical course remains unclear. Therefore, we conducted a cross-sectional analysis of phase III randomized trials for COVID-19 registered at ClinicalTrials.gov.

**Methods:**

We collected the data from ClinicalTrials.gov on March 31, 2021, by specifying the following search conditions under Advanced Search: Condition or disease: (COVID-19) OR (SARS-CoV-2); Study type: Interventional Studies; Study Results: All Studies; Recruitment: Not yet recruiting, Recruiting, Enrolling by invitation, Active, Not recruiting, Suspended, Completed; Sex: All; and Phase: Phase 3. From the downloaded search results, we selected trials that met the following criteria: Primary Purpose: Treatment; Allocation: Randomized. We manually transcribed information not included in the downloaded file, such as Primary Outcome Measures, Secondary Outcome Measures, Time Frame, and Inclusion Criteria. In the analysis, we examined primary and secondary endpoints in trials with severe and non-severe patients, including the types of endpoints, time frame, clinical course, and sample size.

**Results:**

A total of 406 trials were included in the analysis. The median numbers of endpoints in trials with severe and non-severe patients were 9 and 7, respectively. Approximately 25% of the trials used multiple primary endpoints. Regardless of the type of endpoint, the time frames were longer in the trials with severe patients than in the trials with non-severe patients. In the evaluation of the clinical course, worsening was often considered in binary endpoints, and improvement was considered in time-to-event endpoints. The sample size was the largest in clinical trials using binary endpoints.

**Conclusions:**

Endpoints can differ with respect to severity, and the clinical course and time frame are important for defining endpoints. This study provides information that can facilitate the achievement of a consensus for the endpoints in evaluating COVID-19 treatments.

## Background

Since its identification in Wuhan, China, in late 2019, the severe acute respiratory syndrome coronavirus 2 (SARS-CoV-2) has rapidly spread and created a global pandemic of coronavirus disease 2019 (COVID-19). Globally, there have been 149,216,984 confirmed cases of COVID-19 and 3,144,028 deaths as of April 29, 2021 [1]. Most infected patients present with asymptomatic or mild disease [2]. Many patients recover quickly without severe complications, others with severe disease can take 6–8 weeks or longer for recovery, and some patients have a severe form of the disease that can progress to acute respiratory distress syndrome and death [2, 3]. Therefore, urgent development of treatments is needed, especially for patients with severe disease. However, it may be difficult to conduct clinical trials appropriately because there are multiple clinical courses, and there is no consensus on the endpoints in evaluating COVID-19 treatments so far. For studies of any intervention in hospitalized patients with confirmed or suspected COVID-19, the Core Outcome Measures in Effectiveness Trials (COMET) initiative considered mortality and respiratory support as core outcomes [4]. However, they did not mention the definitions of endpoints for these outcomes, such as the types of endpoints and the time frame. In patient populations that are close to recovery, such as patients who do not require oxygen inhalation, the time to recovery is an important endpoint as it is used for evaluating treatments for influenza. However, the COMET initiative did not consider recovery as a core outcome. Therefore, more studies on endpoints in the evaluation of COVID-19 treatments are needed.

Some studies [5–8] have reviewed the endpoints of clinical trials of COVID-19. The endpoints of clinical trials, including randomized and non-randomized trials, involved symptoms, death, recovery, intensive care requirement, hospital discharge, oxygenation, critical illness assessment instruments, and viral load assays [5]. Most clinical trials have used multiple endpoints [5]. Among 49 phase III randomized trials for COVID-19 registered by April 2020, the most common primary endpoint was an ordinal endpoint, which included information such as death, hospitalization, mechanical ventilation, and supplemental oxygen; mortality was the less common primary endpoint [6]. For example, the ordinal scale from 0 (no clinical or virological evidence of infection) to 8 (death), suggested by the World Health Organization (WHO) [9], was used as the primary endpoint [10]. Among all endpoints, including primary and secondary endpoints, mortality was the most common [7]. When there is little information about the illness, treatment, and relevant outcomes, it is difficult to define endpoints [3]. For example, in the adaptive COVID-19 treatment (ACTT-1) study [11], the primary endpoint was changed from an ordinal scale to time-to-recovery after the trial was initiated because of external information that COVID-19 may be more protracted than anticipated.

The challenge of determining endpoints has appeared in randomized trials of remdesivir [10–13]. The primary endpoints were clinical status assessed using the 7-point ordinal scale on day 11 [10], time to recovery [11], time to clinical improvement [12], and in-hospital mortality [13]. The effect on the time to recovery was observed in the overall population (rate ratio 1.29, 95% CI 1.12–1.49); however, the effect was not homogenous among the subjects and was more apparent in patients not requiring supplementary oxygen in the ACTT-1 study [11]. The rate ratio for mortality in the solidarity trial was 0.95 (95% CI 0.81–1.11) [13], and the hazard ratio for mortality through days 14 and 29 in the ACTT-1 study was 0.55 (95% CI 0.36–0.83) and 0.73 (95% CI 0.52–1.03), respectively [11]. These results suggest that endpoints can differ with respect to severity, and the clinical course and time frame are important for the definition of endpoints.

When defining endpoints in clinical trials for COVID-19, several factors should be considered, such as the study population, clinical courses, timing of treatment evaluation, social conditions, clinical importance of the outcome, and feasibility. Treatment effects can vary according to the spectrum of the disease and the timing of treatment [8]. The spectrum of the disease is related to the clinical course, according to which patients will ultimately return to normal function or die even after curative treatment. For example, when defining endpoints, the direction of the clinical courses is related to the handling of competing events. Death is the competing event for time to recovery/improvement, and recovery/improvement is the semi-competing event for time to death [3]. Specifically, the direction of the clinical courses should be considered in endpoints, and endpoints can be different among study populations based on the spectrum of the disease [3]. Although relevant clinical outcomes for COVID-19 may be readily assessed and available within days or weeks [14], a treatment effect that occurs early but dissipates later may not be clinically meaningful, and a treatment effect that occurs later may be missed because of early evaluation [3]. The time frame for endpoints or the timing of evaluation is crucial in a novel disease with substantial heterogeneity [3]. Although there are clinically important endpoints, the use of these endpoints can be challenging because of social conditions and feasibility. For example, ordinal endpoints can become less meaningful when patient numbers exceed hospital capacity, and mechanical ventilators or high-flow oxygen devices are unavailable [3]. The treatment effect on mortality would be clinically meaningful; however, deaths are relatively rare, and few studies would be sufficiently powered to detect the treatment effect [8]. Therefore, the definitions and elements of endpoints remain controversial.

Several challenges are involved in designing clinical trials of treatments for novel infectious diseases, and, as mentioned above, current practices in clinical trials for COVID-19 are unclear. In particular, no study has reviewed the endpoints of clinical trials by considering the factors for defining endpoints and the relationship of endpoints with severity, time frame, clinical course, and sample size. The trial information is reported in the trial registration, and ClinicalTrials.gov, the largest registry of clinical trials globally, provides detailed information. Summarizing the information in registered trials can reveal the definitions of endpoints of clinical interest that can differ between severe and non-severe patients. In general, endpoints differ between exploratory and confirmatory trials; more definitive and more clinically meaningful endpoints are used in confirmatory trials. In this article, we examine and clarify the characteristics of endpoints that reflect clinical interests for the treatment of COVID-19 by using information from phase III randomized trials for COVID-19 registered in ClinicalTrials.gov. In particular, we discuss endpoints that reflect clinical interests by comparing characteristics between clinical trials with severe patients and those with non-severe patients based on combinations of time frame, clinical course, sample size, and types of endpoints.

## Methods

We extracted information on clinical trials for COVID-19 treatments from ClinicalTrials.gov, a database of privately and publicly funded clinical studies conducted around the world. Definitions of data elements in ClinicalTrials.gov are provided and are mostly adapted from 42 CFR Part 11 [15].

A search was conducted at www.ClinicalTrials.gov on March 31, 2021. We specified the following conditions under Advanced Search: Condition or disease: (COVID-19) OR (SARS-CoV-2); Study type: Interventional Studies (Clinical Trials); Study Results: All Studies; Recruitment: Not yet recruiting, Recruiting, Enrolling by invitation, Active, Not recruiting, Suspended, Completed; Sex: All; and Phase: Phase 3. We did not impose restrictions on other conditions. We narrowed the downloaded search results of clinical trials that fit the following criteria: Primary Purpose: Treatment; Allocation: Randomized. For phase II/III studies, only the endpoints in the phase III portion of the trial were included in the analysis. We used the following data from the downloaded search results: Conditions, Interventions, Outcome Measures, Sponsor/Collaborators, Gender, Age, Enrollment (sample size), Study Designs (Primary Purpose), and Locations.

We classified the downloaded search results (locations and interventions) based on whether the trials were conducted in multiple regions and the number of arms was counted. When information for locations was missing, we classified the trial as “unknown.” In some trials, because the arms were not described distinctively in Interventions, a single drug with multiple doses (multiple arms in actual) might be counted as one, and the control treatment not described in interventions was counted as zero. For example, we considered the number of arms to be two when “Other: Placebo|Drug: Remdesivir” was mentioned and one when “Drug: Ivermectin” was mentioned. Although there was only information on a single drug, all trials should have had a control group because we selected trials with “Allocation: Randomized.” Because the number of arms is not directly related to the characteristics of endpoints, and uncertainties may not depend on the types of endpoints, the number of arms was classified based on the observation.

Information on Primary Outcome Measures, Secondary Outcome Measures, and Time Frame were manually transcribed from ClinicalTrials.gov. We classified the clinical courses on Primary Outcome Measures as improvement, when they were related to recovery, improvement, test negative, and loss of detection; worsening, when those were related to death, mortality, progression, worsening, hospitalization; and unknown, when those were neither improving nor worsening. Secondary outcome measures were distinguished as improvement, recovery, and mortality. For example, improvement of two points in ordinal variables and clinical improvement were categorized as improvement, and the time to discharge and proportion of patients exhibiting clinical recovery were categorized as recovery. We verified whether at least one secondary endpoint in each category was used because the number of secondary endpoints was large. We additionally classified the trials according to the type of endpoints (continuous, binary, ordinal, and time-to-event variables). The time frame of each primary endpoint was categorized as 1–14 days (≤14 days), 15–30 days (15–30 days), and more than 30 days (>30 days).

The inclusion of severe patients was defined according to the inclusion criteria in ClinicalTrials.gov. In reference to clinical trials for remdesivir [11, 16], we considered that studies included severe patients when the inclusion criteria contained at least one of the following conditions: (1) requiring invasive or non-invasive mechanical ventilation (or extracorporeal membrane oxygenation (ECMO) or a heart-lung bypass machine), (2) requiring supplemental oxygen, (3) SpO_2_ ≤94% under room air, and (4) tachypnea (respiratory rate ≥24 breaths per minute).

We summarized the characteristics of trials with severe and non-severe patients. The means with standard deviations and medians with interquartile ranges were used for continuous variables, and frequencies and percentages were used for categorical variables. As there was information in all the required fields and “unknown” was used for missing location information, missing values were not considered in the summarizations. We used SAS version 9.4 (SAS Institute, Inc., Cary, NC) for all analyses.

## Results

**Fig. 1:**
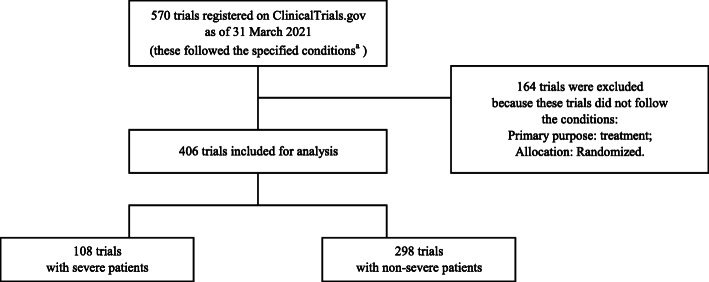
Flowchart identifying trials registered on ClinicalTrials.gov

Among 570 clinical trials selected from ClinicalTrials.gov, 406 were eligible for the analysis (Fig. 1). One hundred sixty-four trials were excluded because these trials did not follow the following conditions: Primary purpose: Treatment; Allocation: Randomized. Table 1 presents the characteristics of the trials.

**Table 1:** Characteristics of eligible clinical trials (*N*=406)

	Severe (*N*=108)	Non-severe (*N*=298)
Region^a^		
Single region	68 (63.0%)	226 (75.8%)
Multi-region	21 (19.4%)	23 (7.7%)
Unknown	19 (17.6%)	49 (16.4%)
Countries^a^		
Canada	4 (3.7%)	19 (6.4%)
USA	35 (32.4%)	58 (19.5%)
France	10 (9.3%)	31 (10.4%)
Germany	8 (7.4%)	9 (3.0%)
Italy	5 (4.6%)	16 (5.4%)
Russia	9 (8.3%)	11 (3.7%)
Spain	11 (10.2%)	18 (6.0%)
UK	11 (10.2%)	13 (4.4%)
China	3 (2.8%)	9 (3.0%)
Japan	6 (5.6%)	6 (2.0%)
Brazil	14 (13.0%)	22 (7.4%)
Others	40 (37.0%)	124 (41.6%)
Number of endpoints		
Mean (SD)	12.8 (10.7)	9.0 (7.7)
Median (IQR)	9 (6, 16.3)	7 (4, 12)
Number of primary endpoints^a^		
1	86 (79.6%)	216 (72.5%)
2	13 (12.0%)	42 (14.1%)
≥3	9 (8.3%)	40 (13.4%)
Number of arms^a^		
1^b^	18 (16.7%)	63 (21.1%)
2^b^	64 (59.3%)	177 (59.4%)
≥3^b^	26 (24.1%)	58 (19.5%)
Sample size (trials with 1 arm^b^)		
Mean (SD)	367.8 (378.2)	396.3 (553.2)
Median (IQR)	205 (100, 462)	200 (100, 466)
Sample size (trials with 2 arms^b^)		
Mean (SD)	519.2 (652.9)	576.9 (831.3)
Median (IQR)	372 (230, 600)	300 (108, 690)
Sample size (trials with ≥3 arms^b^)		
Mean (SD)	806.6 (787.3)	1149.1 (2433.9)
Median (IQR)	475 (200, 1116)	305 (200, 700)

^a^Frequency (percentage); ^b^A single drug with multiple doses that was placed under the same “Arm” in interventions was considered as one arm

*SD* standard deviation, *IQR* interquartile range

There were 108 (26.6%) trials that included severe patients and 298 (73.3%) that did not. Clinical trials with severe patients (hereafter, “Severe”) were conducted in multiple regions more frequently than those with non-severe patients (hereafter, “Non-severe”). Most trials were conducted in the USA (93 trials), and a few trials were conducted in China (12 trials) and Japan (12 trials). Other countries which conducted trials included Egypt (20 trials), Colombia (9 trials), Mexico (8 trials), Pakistan (6 trials), and Argentina (6 trials). The median number of endpoints in “Severe” and “Non-severe” were 9 and 7, respectively; 22 (20.4%) “Severe” trials and 82 (27.5%) “Non-severe” trials used multiple primary endpoints. Two-arm trials (using two drugs) were the most frequent, while approximately 20% of the trials did not describe any control treatment (one-arm trials). The median sample size in “Severe” was larger than that in “Non-severe.”

The characteristics of the primary endpoints in “Severe” and “Non-severe” trials are summarized in Tables 2 and 3, respectively. Multiple primary endpoints were counted individually. Other types of endpoints included safety endpoints. Binary endpoints were mostly used in “Severe” (65 of 153) and “Non-severe” (255 of 481) trials. Regardless of the type of endpoint, the time frames of endpoints used in “Severe” trials were longer because proportions of “≤14 days” were lower. A longer time frame was considered in the time-to-event endpoints than in the other types of endpoints. In the evaluation of the clinical course, regardless of severity, worsening was often considered in the binary endpoints, and improvement was considered in time-to-event endpoints. Worsening in binary and time-to-event endpoints included mortality. In particular, of the 141 trials using a binary endpoint with the direction of worsening as the primary endpoint, 93 trials used mortality. The median sample size was the largest in the clinical trials using binary endpoints.

**Table 2:** Characteristics of primary endpoints in clinical trials with severe patients

	Continuous (*N*=16)	Binary (*N*=65)	Ordinal (*N*=24)	Time-to-event (*N*=44)	Other (*N*=4)
Time frame					
≤14 days	3 (18.8%)	12 (18.5%)	6 (25.0%)	3 (6.8%)	1 (25.0%)
15–30 days	10 (62.5%)	45 (69.2%)	18 (75.0%)	37 (84.1%)	2 (50.0%)
>30 days	3 (18.8%)	8 (12.3%)	0 (0.0%)	4 (9.1%)	1 (25.0%)
Evaluation of clinical course					
Improvement	-	19 (29.2%)	-	34 (77.3%)	-
Worsening	-	39 (60.0%)	-	6 (13.6%)	-
Unknown	-	7 (10.8%)	-	4 (9.1%)	-
Sample size (trials with 1 arm^a^)					
	(*N*=5)	(*N*=10)	(*N*=4)	(*N*=15)	(*N*=1)
Mean (SD)	101.2 (56.4)	472.3 (407.3)	279.3 (226.1)	335.7 (274.5)	180 ( - )
Median (IQR)	76 (76, 76)	239 (197.8, 565.5)	223.5 (173.5, 329.3)	200 (140, 600)	180 ( - )
Sample size (trials with 2 arms^a^)					
	(*N*=6)	(*N*=35)	(*N*=15)	(*N*=22)	(*N*=1)
Mean (SD)	420.7 (175.0)	577.0 (808.8)	476.3 (606.1)	498.4 (320.8)	600 ( - )
Median (IQR)	468 (341.5, 500)	376 (265, 530)	330 (100, 425)	409 (259.5, 677.5)	600 ( - )
Sample size (trials with ≥3 arms^a^)					
	(*N*=5)	(*N*=20)	(*N*=5)	(*N*=7)	(*N*=2)
Mean (SD)	457.8 (498.2)	1124.0 (864.4)	850.0 (1271.8)	1199.1 (733.5)	109.5 (48.8)
Median (IQR)	189 (50, 1000)	1200 (283.5, 1770.5)	450 (100, 500)	1034 (750, 159)	109.5 (-)

^a^A single drug with multiple doses that was placed under the same “Arm” in Interventions was considered as one arm

*SD* standard deviation, *IQR* interquartile range

**Table 3:** Characteristics of primary endpoints in clinical trials with non-severe patients

	Continuous (*N*=68)	Binary (*N*=255)	Ordinal (*N*=71)	Time-to-event (*N*=70)	Other (*N*=16)
Time frame					
≤14 days	35 (51.5%)	79 (31.0%)	39 (54.9%)	11 (15.7%)	5 (31.3%)
15–30 days	26 (38.2%)	144 (56.5%)	30 (42.3%)	44 (62.9%)	8 (50.0%)
>30 days	7 (10.3%)	32 (12.5%)	2 (2.8%)	15 (21.4%)	3 (18.8%)
Evaluation of clinical course					
Improvement	-	61 (23.9%)	-	53 (75.7%)	-
Worsening	-	135 (52.9%)	-	10 (14.3%)	-
Unknown	-	59 (23.1%)	-	7 (10.0%)	-
Sample size (trials with 1 arm^a^)					
	(*N*=24)	(*N*=57)	(*N*=9)	(*N*=16)	(*N*=3)
Mean (SD)	82.6 (99.8)	579.9 (634.8)	290.2 (219.3)	262.3 (303.4)	183.3 (104.1)
Median (IQR)	60 (47.5, 60)	300 (136, 1000)	250 (90, 466)	150 (121, 202.5)	150 (125, 225)
Sample size (trials with 2 arms^a^)					
	(*N*=27)	(*N*=150)	(*N*=44)	(*N*=33)	(*N*=9)
Mean (SD)	730.5 (1675.1)	777.2 (1107.2)	336.8 (238.4)	464.6 (553.7)	1513.6 (2021.3)
Median (IQR)	250 (101, 284)	400 (131.3, 1144.5)	300 (109.5, 480)	278 (100, 554)	1444 (100, 1728)
Sample size (trials with ≥3 arms^a^)					
	(*N*=17)	(*N*=48)	(*N*=18)	(*N*=21)	(*N*=4)
Mean (SD)	530.9 (990.9)	1225.2 (2347.2)	1481.1 (3228.3)	1034.2 (2223.6)	1042.5 (1431.5)
Median (IQR)	200 (200, 200)	320 (200, 1480)	120 (120, 283.5)	320 (200, 676)	475 (247.5, 1270)

^a^A single drug with multiple doses that was placed under the same “Arm” in interventions was considered as one arm

*SD* standard deviation, *IQR* interquartile range

The relationships between the primary and secondary endpoints are presented in Table 4. Regardless of the severity and clinical course of the primary endpoint, both improvement and mortality were evaluated as secondary endpoints. “Severe” trials evaluated more primary and secondary endpoints in the same direction of the clinical course than “Non-severe” trials. Mortality was frequently considered under secondary endpoints as a binary endpoint and was considered more frequently in “Severe” than in “Non-severe” trials. Ordinal variables evaluated longitudinally were used more in “Severe” than in “Non-severe” trials.

**Table 4:** Relationships between primary endpoints and secondary endpoints

	Trials with severe patients			Trials with non-severe patients		
	Primary endpoints			Primary endpoints		
	Improvement^a^ (*N*=45)	Worsening^a^ (*N*=39)	Unknown^a^ (*N*=13)	Improvement^a^ (*N*=97)	Worsening^a^ (*N*=117)	Unknown^a^ (*N*=51)
Secondary endpoints						
Binary						
Improvement	18 (40.0%)	7 (17.9%)	13 (100.0%)	29 (29.9%)	28 (23.9%)	8 (15.7%)
Recovery	3 (6.7%)	2 (5.1%)	13 (100.0%)	5 (5.2%)	2 (1.7%)	51 (100.0%)
Mortality	27 (60.0%)	26 (66.7%)	4 (30.8%)	43 (44.3%)	49 (41.9%)	24 (47.1%)
Time-to-event						
Improvement	20 (44.4%)	14 (35.9%)	5 (38.5%)	29 (29.9%)	31 (26.5%)	8 (15.7%)
Recovery	3 (6.7%)	4 (10.3%)	2 (15.4%)	8 (8.2%)	5 (4.3%)	4 (7.8%)
Mortality	7 (15.6%)	9 (23.1%)	3 (23.1%)	10 (10.3%)	12 (10.3%)	5 (9.8%)
Ordinal						
One time point	9 (20.0%)	6 (15.4%)	2 (15.4%)	10 (10.3%)	20 (17.1%)	6 (11.8%)
Longitudinal	7 (15.6%)	10 (25.6%)	1 (7.7%)	7 (7.2%)	12 (10.3%)	2 (3.9%)

^a^Binary and time-to-event endpoints of primary endpoints

Some clinical trials considered multiple primary endpoints, such as time to recovery and mortality at 28 days, or rate of ventilator use and ordinal variables. Twenty-one trials (20.2%) included ordinal endpoints as one of the multiple primary endpoints. Additionally, among the clinical trials that evaluated binary endpoints or time-to-event as the primary endpoint, 21.2% (21 of 99) evaluated both directions of clinical course (improvement and worsening). Finally, there were trials in which competing risk problems could occur, such as mortality (binary) and discharge (time-to-event), mortality (binary) and receiving invasive mechanical ventilation or ECMO (binary), or mortality (time-to-event) and recovery (time-to-event). When severity was not distinguished, the median sample sizes in trials with multiple primary endpoints and in those with a single primary endpoint were 310 and 300, respectively. This tendency was observed for both “Severe” and “Non-severe” trials.

## Discussion

This study summarized the characteristics of phase III randomized trials for COVID-19 in ClinicalTrials.gov. Among phase III randomized trials for COVID-19 in the International Clinical Trials Registry Platform (ICTRP) [17], which were updated on April 13, 2021, 72.5% of the trials were registered at ClinicalTrials.gov, and 24.1% of the trials were registered in the Iranian Registry of Clinical Trials (IRCT). All trials registered in the IRCT were conducted in Iran, and the median target sample size was only 70 patients (data not shown). Therefore, our results are representative because they are based on large clinical trials conducted in various countries and registered at ClinicalTrials.gov. Because the downloaded file from the IRCT did not include enough information for our analysis, we did not use data from the IRCT. In the analysis, we focused on the types, time frame, and clinical course of the endpoints based on severity. We also evaluated the relationships between the primary endpoints and the sample size and the secondary endpoints. Although some researchers [3, 18, 19] have proposed new endpoints for evaluating COVID-19 treatments, it is necessary to review the endpoints that have been used in practice to reach a consensus for the evaluation of COVID-19 treatment. This study provides information that can facilitate this consensus.

According to this survey of trials registered in ClinicalTrial.gov, approximately 25% of the trials used multiple primary endpoints. This is partly because COVID-19 is a novel infectious disease, and an evaluation method has not yet been established. Binary endpoints were the most common primary endpoints. These results largely differed from those of a previous study conducted by April 2020 [6]. One of the reasons for this difference is that mortality was considered a binary endpoint (whether a patient survived across a specific time window) rather than a time-to-event endpoint (time to death from randomization). If treatment aims to prevent mortality for the entire period in short-term studies, binary endpoints may be more clinically relevant [20, 21]. Although the sample size of clinical trials using a binary endpoint was larger in this survey, which may be due to the loss of information on binary endpoints compared to time-to-event endpoints, these trials could have considered clinical relevance as well as development speed. On the other hand, considering hospital capacity and availability of mechanical ventilators or other devices, evaluation of mortality by time-to-event endpoints, as in the ACTT-1 study [11] and the Solidarity trial [14], may be meaningful. Ordinal variables were not only used directly as the primary endpoint but also used for the definition of binary and time-to-event endpoints (recovery or improvement). The reasons to avoid using an ordinal endpoint may be as follows: it is difficult to interpret the results of analysis methods such as the proportional odds model, the power in using the time-to-event endpoint could be larger than that with the ordinal endpoint [3], and analyzing longitudinal ordinal endpoints among survivors can lead to bias (survivor bias) [18].

The time frames of the endpoints were longer in clinical trials with severe patients, which may be attributed to the relationship between the clinical course and the severity. In the ACTT-1 study, the time to recovery in non-severe patients was shorter than that in severe patients [11]. According to this survey, mortality was evaluated as a secondary endpoint more frequently in clinical trials with severe patients than in those with non-severe patients. To appropriately evaluate mortality, a longer time frame may be required. The time frame of endpoints other than mortality may be determined by considering that of mortality. Although the time point of primary interest for evaluating each endpoint could be different, these time points may be related because each endpoint can be a competing event [3]. When interpreting the results of competing risk analysis, it is important to show not only the endpoint of interest but also the endpoints of the competing event [22, 23].

In clinical trials for COVID-19, several clinical courses would need to be evaluated. Although worsening was evaluated using binary endpoints and improvement was evaluated by a time-to-event endpoint, according to the survey, there is no rationale for using a particular endpoint. Longitudinal evaluation is necessary when the clinical course can be complex. The definitions of improvement and recovery varied among the trials. When using improvement of one or more categories from the baseline category as the definition, it is assumed that the clinical meaningfulness of the improvement does not depend on the baseline category. However, for example, there is no guarantee that improvement from 5 to 3 and from 4 to 2 present the same meaning when using the WHO ordinal scale [9]. Therefore, it would be better to consider the baseline status or the threshold for improvement. In at least half of the trials, both directions of clinical courses were evaluated, regardless of the primary and secondary endpoints. Evaluating both directions in longitudinal changes can result in analyses with moderate to good power [24]. In clinical trials for infectious diseases, both directions of the clinical course may be considered important in both clinical and statistical aspects.

Based on this survey, we suggest definitions of endpoints for novel infectious diseases. When treatment aims to prevent mortality for the entire period in short-term studies, a binary endpoint should be used [20, 21]. However, in the early stage of the pandemic, it may be possible to use time-to-event endpoints in consideration of hospital capacity, which may cause censoring. In this case, it should be better to evaluate the treatment effect based on survival probabilities at a specific time point rather than the hazard ratio. Although the time frame for mortality in non-severe patients could be shorter than that in severe patients, at least 14 days should be considered. When treatment aims to improve recovery, a time-to-event endpoint should be used because it is important to shorten illness duration. However, a soft definition of improvement (recovery), such as improvement of two points in ordinal variables, may not be meaningful for the time-to-event endpoint because some patients who initially required high-flow oxygen may later require ECMO. “No need for treatment” such as disappearance of symptoms or discharge would be a better definition of improvement. However, we should be careful about reasons such as exceeding hospital capacity [3] when discharge is used for the definition, especially during the pandemic. Although the time frame for non-severe patients could be shorter than that for severe patients, it depends on the definition of improvement. In the early stage of the pandemic of a novel infectious disease, it is difficult to determine appropriate endpoints for treatments; however, we could choose appropriate endpoints based on information from the clinical courses of early patients. For COVID-19, severity or baseline characteristics should be considered when choosing endpoints.

Discussions on the results of this survey are related to some attributes of estimand [25], population, variable (endpoint), and intercurrent events. In this situation, the severity is related to the population. Death (mortality) can be either an endpoint or an intercurrent event. The intercurrent events to be considered depending on the estimand. The way death is handled may vary depending on the strategy. For example, when time to ventilation and time to death are endpoints (both endpoints are related to “worsening” events), we can consider a composite variable strategy (time to first event). When recovery and death are endpoints (one is related to “improvement,” the other is related to “worsening”), it could be better to apply the competing risk approach. The hypothetical strategy and the principal stratum strategy may be inappropriate in this situation because the assumption that death would not occur would not be realistic, and the principal stratum of always survivors would not be a target population. In this study, we had access only to the information in ClinicalTrials.gov, so we could not ascertain the estimand of each trial.

This study has some limitations. First, in this survey, severity may differ between trials with severe patients. For example, some trials might include patients requiring small amounts of supplemental oxygen, and other trials might include those requiring ECMO. Thus, the choice of endpoints depends on the detail of severity. Second, information on improvement (recovery) may not be sufficient. As discussed, the definition of improvement can affect the time frame. In addition, we did not mention the details of continuous endpoints. Finally, we did not extract information on the analysis methods related to the selection of endpoints and sample size. For example, a competing risk analysis was considered in the ACTT-1 study [11]. This information may be obtained from published articles of clinical trials; however, it was difficult to extract this information from ClinicalTrials.gov. The statistical properties of the endpoints should be evaluated by simulation studies based on actual clinical trials, such as the ACTT-1 study. Based on the present survey results, we will propose a new endpoint for evaluating the clinical course in both directions and compare it with existing endpoints in a future study.

## Conclusions

In this survey, we found that many endpoints, multiple primary endpoints, binary endpoints for worsening, and time-to-event endpoints for improvement were considered in the registered clinical trials for COVID-19. The characteristics of the endpoints in COVID-19 treatment trials depended on whether patients with severe disease were included. Although challenges remain, this survey provides information that can facilitate the achievement of a consensus for endpoints in evaluating COVID-19 treatments.

## Data Availability

All data are available for academic researchers upon request.

## Abbreviations

ACTT-1: Adaptive COVID-19 treatment (ACTT-1)
COMET: Core Outcome Measures in Effective Trials
COVID-19: Coronavirus disease 2019
ECMO: Extracorporeal membrane oxygenation
ICTRP: International Clinical Trials Registry Platform
IRCT: Iranian Registry of Clinical Trials
SARS-CoV-2: Severe Acute Respiratory Syndrome Coronavirus 2
WHO: World Health Organization
